# Minimal encephalopathy in hereditary hemorrhagic telangiectasia patients with portosystemic vascular malformations

**DOI:** 10.1186/s13023-024-03493-3

**Published:** 2024-12-21

**Authors:** B. Villanueva, A. Cañabate, R. Torres-Iglesias, P. Cerdà, E. Gamundí, Q. Ordi, E. Alba, L. A. Sanz-Astier, A. Iriarte, J. Ribas, J. Castellote, X. Pintó, A. Riera-Mestre

**Affiliations:** 1 HHT Unit. Hospital Universitari Bellvitge, C/Feixa Llarga S/N. L’Hospitalet de Llobregat, 08907 Barcelona, Spain; 2 Internal Medicine Department, Hospital Universitari Bellvitge, Barcelona, Spain; 3 Bellvitge Biomedical Research Institute (IDIBELL), Barcelona, Spain; 4 Internal Medicine Department, Hospital Universitari Son Espases, Mallorca, Spain; 5 Cytology and Hematology Laboratory, Anatomic Pathology Department, Hospital Universitari Bellvitge, Barcelona, Spain; 6 Angioradiology, Radiology Department, Hospital Universitari Bellvitge, Barcelona, Spain; 7 Pneumology Department, Hospital Universitari Bellvitge, Barcelona, Spain; 8 Department of Digestive Diseases, Hospital Universitari Bellvitge, Barcelona, Spain; 9 Clinical Sciences Department, Faculty of Medicine and Health Sciences, Universitat de Barcelona, Barcelona, Spain; 10 Center for Biomedical Research in Obesity and Nutrition Physiopathology Network (CIBEROBN), Carlos III Health Institute, Madrid, Spain

**Keywords:** Hereditary hemorrhagic telangiectasia, Hepatic encephalopathy, Rare diseases, Portosystemic malformations

## Abstract

**Background:**

Hereditary hemorrhagic telangiectasia (HHT) is characterized by telangiectasia and larger vascular malformations. Liver malformations are the most frequent visceral involvement including the presence of portosystemic malformations (PSM) that can cause hepatic encephalopathy. Minimal hepatic encephalopathy (mHE) is characterized by alterations of brain function in neuropsychological or neurophysiological tests and decreases quality of life. The evidence of mHE in HHT patients is scarce. The aim of this study is to assess the prevalence and health impact of mHE in patients with and without PSM.

**Methods:**

We performed a cross-sectional observational study in a cohort of patients from an HHT referral unit. Adult patients with definite HHT and PSM and age and sex matched HHT controls without PSM (1:1) were included. Baseline clinical, imaging and laboratory tests and different neuropsychological tests for the screening of mHE were compared between both groups.

**Results:**

Eighteen patients with PSM and 18 controls out of 430 HHT patients were included. Patients with PSM showed higher prevalence of attention disturbances (50% vs. 11.1%, *p* = 0.027), falls during last 12 months (22.2% vs. 5.6%, *p* = 0.338), sleep disorders (50% vs. 16.7%, *p* = 0.075) and a worst performance in s-ANT1 test (14 vs. 19.5 points score, *p* = 0.739) than HHT controls.

**Conclusions:**

HHT patients with PSM showed higher attention difficulties than HHT controls, though both PSM and HHT controls showed findings of mHE. Specific neuropsychological tests for early detection of mHE should be considered in HHT patients.

## Background

Hereditary hemorrhagic telangiectasia (HHT) or Rendu-Osler–Weber syndrome is an autosomal dominant genetic disorder classified as a rare disease (ORPHA: 774) with an incidence of 1/6000 [1]. HHT can be diagnosed using either the Curaçao criteria or through molecular genetic testing [2]. Pathogenic variants in endoglin (*ENG*) and activin A receptor like type 1 (*ACVRL1*) genes are detected in more than 85% of patients submitted to molecular diagnosis and cause HHT type 1 and type 2, respectively [3, 4]. Endoglin (encoded by *ENG*) is an auxiliary co-receptor at the endothelial cell surface that promotes BMP9/10 signaling through the activin receptor-like kinase 1 receptor (ALK1; encoded by *ACVRL1*) [5]. Together, they contribute to the activation of endothelial BMP/SMAD signaling and are involved in the counter regulation of angiogenesis. Thus, loss-of-function variants result in abnormal vascular growth leading to telangiectases and larger vascular malformations (VM) [6].

Hepatic VM are the most frequent visceral involvement in HHT and have been described in up to 75–84% of patients, mostly in HHT type 2 [4, 7, 8]. In fact, HHT is the most common cause of congenital liver VM in adults [9]. Although mostly asymptomatic, some patients with hepatic VM develop severe clinical symptoms and require liver transplantation (LT) [10]. There are three types of hepatic vascular shunting in HHT patients: arteriovenous (hepatic artery to hepatic veins), arterioportal (hepatic artery to portal vein) and portosystemic malformations (PSM, portal vein to hepatic veins). These three types of VM can cause high-output cardiac failure (HOCF) and/or ischemic cholangitis, portal hypertension or hepatic encephalopathy (HE), respectively [7, 8]. Despite one of these three different clinical pictures can predominate, they usually coexist and show fluctuation and transition from one to another [7, 8, 11].

HE is a potentially reversible disorder characterized predominantly by alterations of personality, consciousness, cognition and motor function produced by liver insufficiency and/or portal-systemic blood shunting [12, 13]. HE in HHT patients is infrequent, though it is probably underdiagnosed [8, 14, 15]. Despite this, in four out of 83 HHT patients from the largest series of HHT patients who underwent LT, HE was described as the main indication for LT (with HOCF symptoms in three patients and ischemic cholangitis in another one) [10]. The West Haven criteria are the most frequently tool used for grading HE and includes four grades of clinically manifest HE and one grade of minimal HE (mHE) [13].

Although there are not universal diagnostic criteria for mHE, it is characterized by alterations of brain function in neuropsychological or neurophysiological tests without clinical signs of HE [12, 16]. Despite its subclinical nature, mHE affects quality of life, executive functions or areas of attention, causes sleep disorders, predisposes to clinical HE and reduces life expectancy [17]. However, mHE is poorly assessed in clinical practice, so it is probably under-recognized and under-diagnosed [18–20]. The evidence on the prevalence of mHE in patients with HHT is scarce and there is no evidence related to the presence of hepatic shunt. [21]. More objective data are needed to improve early diagnosis of HE in a disease such as HHT with a high prevalence of hepatic vascular involvement. The aim of this study is to assess the prevalence and impact of mHE in HHT patients according to the presence of PSM.

## Methods

### Study design

This is an observational cross-sectional study performed on a prospective cohort that includes all consecutive patients attended at a referral HHT Unit in a university hospital from September 2011 to December 2023. Patients with a definite HHT diagnosis according to the Curaçao criteria (meeting ≥ 3 criteria) or a positive genetic test, and objectively confirmed PSM at computed tomography (CT) were included as cases. For the control group, all included cases were matched by age and sex in a 1:1 ratio with HHT patients from the same cohort but without PSM. All of these patients had an abdominal CT examined by a trained radiologist without PSM in the previous 5 years. Exclusion criteria were: liver cirrhosis or chronic liver disease, transjugular intrahepatic portosystemic shunt (TIPS), end-stage cardiac, respiratory or renal comorbidities, hyponatremia (sodium levels lower than 125 mmol/l) or neurological diseases including dementia.

All patients provided consent to participate in the study according to the local Clinical Research Ethics Committee requirements. We followed the Strengthening the Reporting of Observational Studies in Epidemiology (STROBE) statement guidelines for observational cohort studies [22]. Personal and clinical data collected for the study are in line with the Spanish Data Protection Act (*Ley Orgánica 3/2018 de 5 de diciembre de Protección de Datos Personales*). The study was approved by the Clinical Research Ethics Committee of the Hospital Universitari de Bellvitge (approval number PR018/24).

The main objective was to compare easy to apply diagnostic tests and symptoms related to mHE in HHT patients according to the presence or absence of PSM at abdominal CT.

### Clinical variables

Baseline demographic data, underlying diseases, Curaçao criteria, Epistaxis Severity Score (ESS), genetic testing, anemia-related parameters, liver and kidney function laboratory tests and level of education were collected. ESS is an online tool that quantifies epistaxis severity considering different parameters during the previous three months [23]. All laboratory test and EES evaluation were performed during the 12 months before the mHE evaluation period. Screening for HHT-related visceral involvement is described elsewhere and includes an initial screening abdominal CT in all patients to define the subtype of liver shunting involvement and the consequent required follow-up [8]. Central nervous involvement screening with CT or magnetic resonance imaging (MRI) screening is recommended in those patients with family history of brain VMs and/or with neurological symptoms, but not in all adult HHT patients [24]. All radiological tests were reviewed by two expert radiologists in HHT (Q.O. and E.A.).

### mHE evaluation

All patients included were evaluated by telephone. They were asked for subjective attention deficiency (yes or no), sleeping disorders (yes or no), and falls during the last 12 months (any fall/no falls). The simplified Animal Naming test (s-ANT1) was also used for the assessment of mHE. In this test, subjects are asked to name as many animals as possible within one minute. A score of ≥ 15 indicates a probability of about 80% that the subject has no cognitive dysfunction and a score of < 10 indicates the presence of encephalopathy with a probability of about 80% [12, 25]. Short Portable Mental State Questionnaire (Pfeiffer scale) was also performed in all patients to rule out patients with cognitive impairment [26]. The rationale for selecting these tests is mainly the easiness to apply in the daily clinical practice, even in the telemedicine scenario, based on the evidence to detect mHE symptoms in cirrhotic patients [17, 25]**.**

### Statistical analysis

A descriptive statistical analysis was performed for categorical and continuous variables and expressed as proportions or means with standard deviations (SD). Baseline clinical characteristics and outcomes were compared between patients with and without PSM. Categorical variables were compared with the Chi-square test or the Fisher exact test, whereas the t-test was used to compare continuous variables. The Kolmogorov–Smirnov test was performed for continuous variables to assess normality. Non-parametric variables were expressed as median (interquartile range -IQR-) and compared with the U Mann–Whitney test. A value of *p* < 0.05 was considered statistically significant. Analyses were performed using IBM SPSS Statistics, version 22.0 for the PC (IBM Corp., Armonk, NY, USA).

## Results

**Fig. 1:**
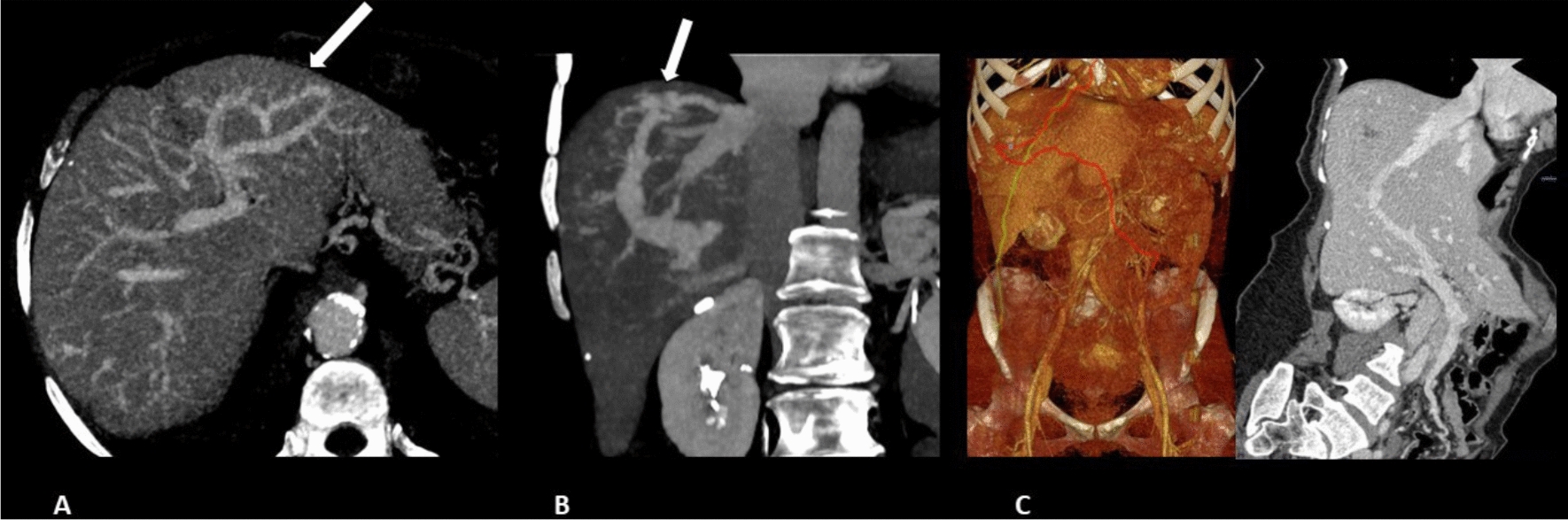
**A** Axial MIP (maximum intensity projection) computed tomography (CT) image obtained in the hepatic phase showing portohepatic venous shunting in the left hepatic lobe. **B** Coronal MIP CT image showing portohepatic venous shunting, with dilated portal vein communicating the right hepatic vein through a focal vascular mass. **C** 3D CT reconstruction showing portovenous shunting with the right hepatic vein

Twenty-three (5.6%) out of 410 patients attended at our HHT referral Unit showed PSM. Among them, 5 were excluded due to dementia (1), liver cirrhosis (1), lost of follow-up (2) or not agree to participate (1). Finally, 18 patients with PSM and 18 matched control HHT patients were included. An example of PSM is shown in Fig. 1**.** Overall, 77.8% were women with a mean age of 64.1 years. HHT patients with PSM and controls did not show statistically significant differences in clinical or laboratory test results except for higher liver involvement prevalence (100% vs 73.9%; *p* = 0.019), higher AST levels (24.4 ± 5.9 vs 19.1 ± 7.1, *p* = 0.018) and higher atrial fibrillation prevalence (33.3% vs 0%, p = 0.019). Demographic, clinical and laboratory characteristics from both groups are shown in Table 1.

**Table 1:** Clinical characteristics and laboratory tests in HHT patients with PSM (HHT-PSM) and HHT control without PSM groups

		HHT controls (n=18)	HHT-PSM (n=18)	*p*
Age (years), mean (SD)		62.7 (11.1)	65.61 (11.9)	0.445
Smokers, n (%)	Yes	6 (33.3)	3 (16.6)	0.424
	No	10 (55.6)	11 (61.7)	
	Previous	2 (11.1)	4 (22.2)	
Arterial hypertension, n (%)		11 (61.1)	8 (44.4)	0.505
Diabetes, n (%)		2 (11.1)	3 (16.7)	1.000
Dyslipidemia, n (%)		4 (22.2)	5 (27.8)	1.000
Previous CVA, n (%)		3 (16.7)	0 (0.0)	0.229
Atrial fibrillation, n (%)		0 (0.0)	6 (33.3)	0.019
Previous overt HE, n (%)		0 (0.0)	2 (11.1 )	0486
Education level, n (%)	Primary studies	11 (61.1)	9 (50.0)	0.766
	Secondary studies	2 (11.1)	2 (11.1)	
	University	5 (27.8)	7 (38.9)	
Genetics, n (%)	*ENG*	9 (50.0)	5 (27.8)	0.294
	*ACVRL1*	6 (33.3)	10 (55.6)	
	Negative	1 (5.6)	2 (11.1)	
Visceral involvement				
Lung involvement, n (%)		7 (88.9)	5 (27.8)	0.725
Liver involvement, n (%)		12 (66.7)	18 (100.0)	0.019*
Cardiac index (L/min/m^2^), mean (SD)		2.90 (0.7)	3.33 (0.7)	0.083
GI involvement, n (%)		10 (55.6)	10 (55.6)	1.000
CNS involvement, n (%)		2 (11.1)	1 (5.6)	1.000
ESS, mean (SD)		1.01 (0.6)	0.90 (0.9)	0.664
Blood test				
Hemoglobin, mean (SD)		120 (20.6)	132 (17.9)	0.071
Ferritin, median (IQR)		33.5 (21.1–93.7)	83.0 (45.5–174.9)	0.460
TS, median (IQR)		17.5 (13.0–26.3)	19.0 (14.5–28.8)	1.000
Bilirubin, median (IQR)		7 (5.0–7.0)	7 (6.0–12.3)	0.737
AST, mean (SD)		19.1 (7.1)	24.4 (5.9)	0.108*
ALT, median (IQR)		17.5 (13.0–26.3)	15.0 (11.0–24––5)	0.182
GGT, median (IQR)		23.5 (15.8–86.0)	33.0 (23.3–53.3)	0.739
AF, median (IQR)	92 (72––115)	91 (66–125)	0.869	0.869
INR, median (IQR)	1.03 (0.98–1.10)	1.05 (0.99–1.19)u	0.731	0.731
Albumin, mean (SD)	44.62 (4.8)	44.00 (4.2)	0.695	0.695
Creatinine, median (IQR)	65.7 (59.5–79.3)	67.0 (61.5––78.0)	1.000	1.000
Pfeiffer score, n (%)	0	13 (72.2)	7 (38.9)	0.118
	1	3 (16.7)	8 (44.4)	
	2	2 (11.1)	3 (16.7)	

^*^ indicates *p* < 0.05

ACVRL1, activin A receptor like type 1 gene; AF, alkaline phosphatase; ALT, alanine aminotransferase; AST, aspartate aminotransferase; CNS, central nervous system; CVA, cerebrovascular accident; EES, epistaxis severity score; ENG, endoglin gene; GI: gastrointestinal; GGT, Gamma-glutamyltransferase; HE, hepatic encephalopathy; IQR, interquartílic range; INR, international normalized ratio; PSM; portosystemic malformations; SD: standard deviations; TS, transferrin saturation

**Fig. 2:**
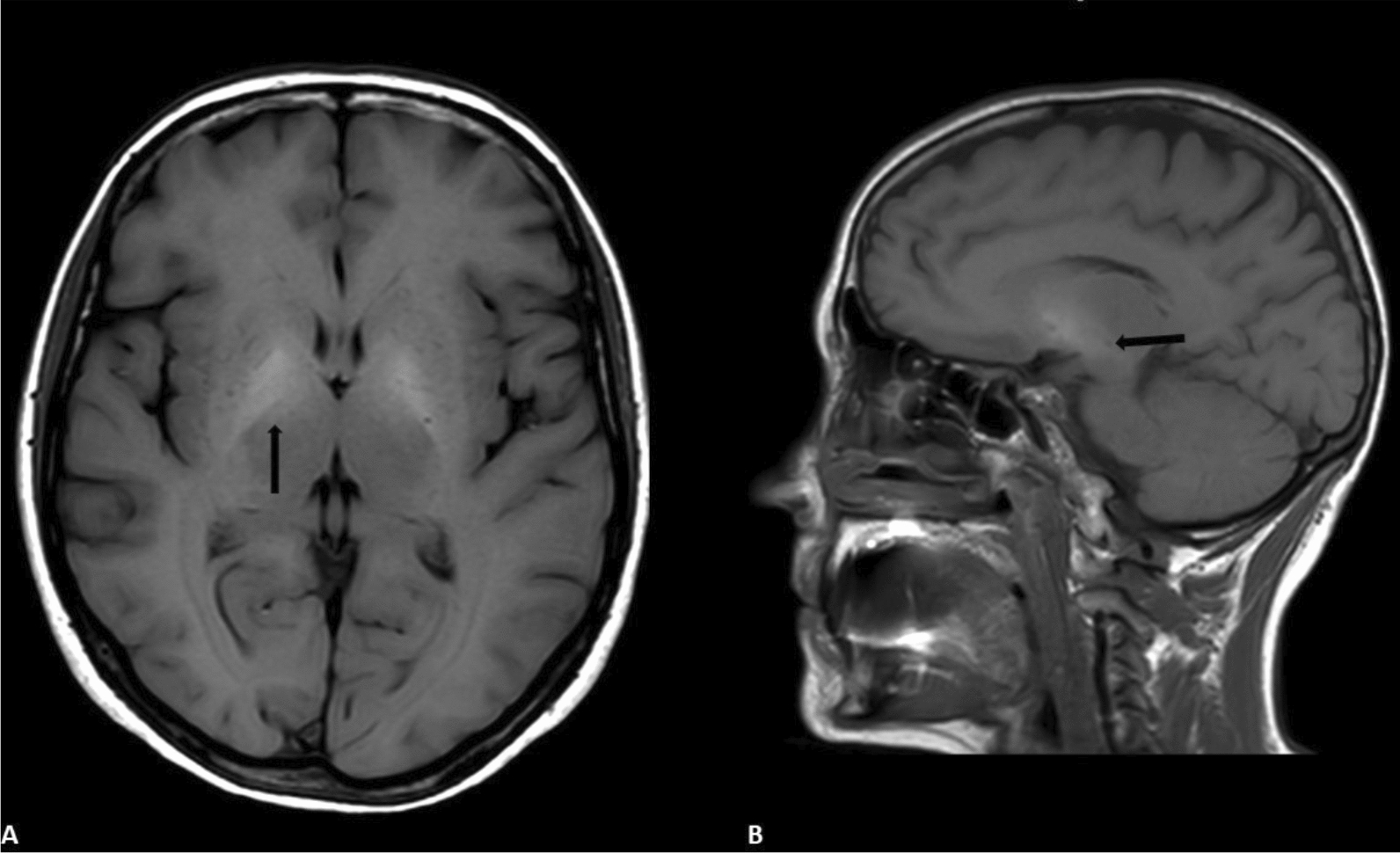
T1-weighted imaging showing high signal intensity in the basal ganglia without mass effect, which results from paramagnetic manganese deposition (**A**: axial plane;** B**: sagital plane)

Five controls and 11 PSM patients had a screening brain MRI; two patients in the control group and one in the PSM group showed vascular brain involvement. Brain manganese deposition (see an example in Fig. 2) at MRI was similar in both control and PSM groups, one out of five (20%) and four out of 11 (36.4%) patients, respectively.

**Fig. 3:**
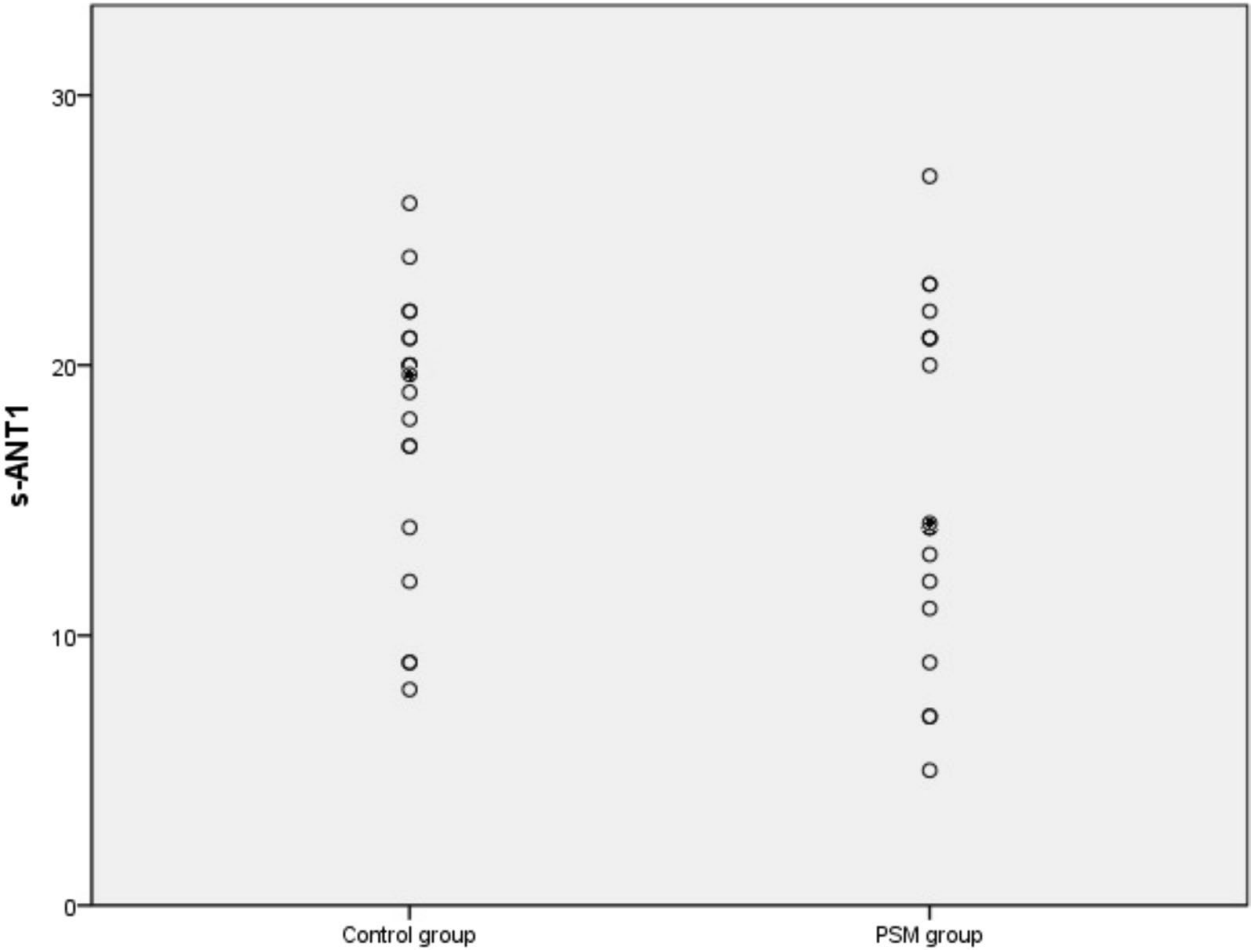
Distribution of s-ANT1 results between PSM and control group. Abbreviations: PSM, portosystemic malformation; s-ANT1, Simplified Animal Naming Test

Regarding neuropsychological or neurophysiological tests, 50% of patients with PSM and 22.2% of controls achieved less than 15 points at the s-ANT1 test (*p* = 0.164). Using the 10 point cutoff 2 control patients (11.1%) and 4 PSM patients (22.2%) achieved less than 10 points. The s-ANT1 results between both groups are shown in Fig. 3**.** Nine patients in the PSM group (50%) and three in the control group (16.7%) reported sleep disturbances in the last year (*p* = 0.075). One patient in the control group (5.6%) and four in the PSM group (22.2%) reported at least one fall during the last year (*p* = 0.338). Finally, 2 patients reported attention disturbances in the control group (11.1%) in contrast with 9 patients in the PSM group (50%, *p* = 0.027) during the last year (Table 2).

**Table 2:** Neuropsychological characteristics comparing PSM and control group

	Controls	PSM	p
s-ANT1 Absolute, median (IQR) < 15, n (%) < 10, n (%)	19.5 (13.5–21.6) 4 (22.2) 2 (11.1)	14 (10.5–21.3) 9 (50.0) 4 (22.2)	0.739 0.164 0.658
Sleep disorder, n (%)	3 (16.7)	9 (50.0)	0.075
Falls (last 12 months), n (%)	1 (5.6)	4 (22.2)	0.338
Atention disturbances, n (%)	2 (11.1)	9 (50.0)	0.027*

^*^ indicates *p* < 0.05

IQR, interquartílic range; s-ANT1, simplified animal naming test; PSM, portosystemic malformations; SD, standard deviation

We also aimed to compare all the tests according to the presence of manganese deposition at brain MRI, finding no differences amongst both groups (Table 3).

**Table 3:** Neuropsychological characteristics comparing patients with and without manganese deposit finding at brain MRI

	Positive MRI (n = 5)	Negative MRI (n = 11)	*p*
s-ANT1 Absolute, median (IQR) < 15, n (%) < 10, n (%)	21 (10.5–22.0) 2 (40) 1 (20)	14.0 (12.0–21.0) 4 (40) 1 (10)	0.583 1.000 1.000
Sleep disorder, n (%)	1 (20)	5 (50)	0.580
Falls (last 12 months), n (%)	0 (0)	3 (30)	0.505
Atention disturbances, n (%)	1 (20)	5 (50)	0.580

IQR, interquartílic range; s-ANT1, simplified animal naming test; MRI, magnetic resonance imaging; SD, standard deviation

## Discussion

To the best of our knowledge, this is the first study assessing mHE in HHT patients according to the presence of PSM at abdominal CT. In our study, patients with PSM showed a statistically significant higher prevalence of attention deficiency than HHT controls without PSM, and a worst performance in the rest of applied tests, though not reaching statistical significance. Attention deficiency has been related to mHE through psychometric tests by detecting dysfunction across allthree attention subsystems: vigilance, orienting and executive functions [27]. In addition, attention deficiency significantly impacts on quality of life, asit affectsdaily functioning and overall well-being. Patients could experience difficulties with simple tasks that require sustained attention like driving, working, or managing daily common responsibilities [28].

*Barone *et al. explored the presence of mHE in 37 unselected HHT patients by firstly performing critical flicker frequency (CFF) and then the s-ANT1 test in patients with abnormal CFF value [21]. Patients were considered as subclinical neurological alterations if both tests resulted abnormal (which occurred in four patients). Arterio-venous shunts were reported in 30 out of 37 patients, but no patient had PSM. The s-ANT1 normalization after lactulose administration in all four patients with mHE lead the authors to consider that mHE could be caused by a liver by-pass secondary to radiologically undetectable portosystemic microfistulas. Despite the evidence about the benefit of treatment with lactulose for mHE manifestations in cirrhotic patients, the evidence of this therapy in HHT is very scarce [21, 29, 30].

Despite mHE represents the earliest and mildest form of HE, it is under-diagnosed. Probably the main reason is the doubts about which test should be used, but also the uncertainty about the test’s ability to identify patients who will benefit from treatment [19, 20, 31]. If these reasons mostly impact in clinicians dealing with cirrhotic patients, hesitation and mHE tests are more common in other infrequent diseases, such as HHT. Thus, mHE diagnosis is more difficult in these uncommon scenarios than in cirrhosis because symptoms may resemble those of other more common neuropsychiatric conditions. In fact, there is no current consensus on how mHE should be diagnosed and it is mostly based on excluding other causes of altered mental status. Thus, there is a need of a high clinical suspicion of mHE in HHT and other diseases with liver function impairment or porto-systemic shunting, and to engage clinicians in testing for early diagnosis of this condition.

Interestingly, a female predominance was observed in patients with PSM (70.8%). In fact, women showed severe liver involvement both in HHT1 and HHT2 patients [32]. This worst situation is also seen in the largest published series of HHT patients with LT, where 81.9% of 83 patients were women [10]. In PSM group a slightly higher AST levels were found, probably related to a higher liver involvement in these patients. Moreover, atrial fibrillation was significantly more frequent in the PSM group. This finding could also be related with higher liver involvement in these patients. In HHT, different types of vascular shunting usually coexist including arteriovenous shunts, with the subsequent previously reported cardiac impact resulting in HOCF and atrial fibrillation [7]. This explanation is supported by the higher cardiac index in the PSM group, even though not reaching statistical significance. No overt ischemic episodes that could have influenced in the mHE development were detected in the six patients with atrial fibrillation from the PSM group. In fact, although evidence suggests a positive association between atrial fibrillation and cognitive impairment, the role of atrial fibrillation needs to be confirmed in larger and longer prospective cohort studies that use precise neuropsychological and cognitive function assessments [33].

Notably, a high percentage of patients in our cohort presented with neuropsychological disorders, even those without PSM. For example, sleeping disorders were present in 30.5% of all patients while the prevalence of chronic insomnia is approximately 10% of the adults in industrialized European countries, with an age-related increase in prevalence rates [34]. This higher prevalence could also be as the result of the presence of microscopic PSM not visible in CT scan as proposed by *Barone *et al. [21].

Five patients showed basal ganglia manganese deposition at MRI tests, all of them had any kind of HHT liver involvement with a predominance in the PSM group. This finding is well described in HHT patients as a result of the presence of hepatic shunts allowing the manganese to escape into systemic circulation [35–37]. It has been related to *ACVRL1* gene mutation, older age and the presence of cirrhosis or iron-deficiency anemia [38]. In our study three out of five patients had ACVRL1 mutation (one had *ENG* mutation and one was not tested), only one had anemia and the mean age was not comparable due to matching. The presence of manganese deposition in patients without macroscopic PSM supports also the presence of microvascular portosystemic shunts. Moreover, it discourages to use MRI as a screening for mHE in HHT patients because of the absence of correlation between anatomic findings and clinical symptoms.

The present study has several limitations. First, it is carried out in an HHT reference center with expert HHT radiologists that are trained to detect PSM, not commonly specified in other scenarios, and differentiate them from other liver VM. Second, the study lacks of any objective test to detect mHE and the used ones could be modified by other factors not related to mHE, for this reason we adjusted for confounding factors that may influence in cognitive performance like age or education level and we excluded dementia and overt HE at the moment of the evaluation to minimize this issue. However, there are other unaddressed factors that could influence this relationship such as psychoactive or opioid medications. Third, the limited number of included patients is a common challenge when dealing with patients with rare diseases, even more when facing uncommon complications of these diseases, such as PSM in HHT; it limits the generalizability of our results in other scenarios. Finally, this is a cross-sectional study lacking follow-up of these patients.

## Conclusions

HHT patients with PSM showed higher attention difficulties than HHT controls, though both PSM and HHT controls showed findings of mHE. The performance of specific neuropsychological tests for early detection of mHE should be considered in HHT patients. The usefulness of the presence of brain manganese deposition at MRI and the potential benefit of purgative or other therapies for mHE in HHT patients, needs further research.

## Data Availability

The raw datasets generated and/or analyzed during the current study are not publicly available due to personal data restrictions but are available from the corresponding author on reasonable request. This excludes any individual personal/clinical data of the individuals, which would endanger their anonymity.

## Abbreviations

ACVRL1: Activin A receptor like type 1
AF: Alkaline phosphatase
ALT: Alanine aminotransferase
AST: Aspartate aminotransferase
AVMs: Arteriovenous malformations
CCF: Critical flicker frequency
CI: Confidence intervals
CT: Computed tomography
HE: Hepatic encephalopathy
ENG: Endoglin
ESS: Epistaxis severity score
GGT: Gamma-glutamyltransferase
GI: Gastrointestinal
HHT: Hereditary hemorrhagic telangiectasia
HOCF: High-output heart failure
INR: International normalized ratio
LT: Liver transplantation
mHE: Minimal hepatic encephalopathy
MRI: Magnetic resonance imaging
PSM: Portosystemic malformations
SD: Standard deviations
STROBE: Strengthening the Reporting of Observational Studies in Epidemiology
TIPS: Transjugular intrahepatic portosystemic shunt
TS: Transferrin saturation
VM: Vascular malformation
